# Independent Evolution of Leaf and Root Traits within and among Temperate Grassland Plant Communities

**DOI:** 10.1371/journal.pone.0019992

**Published:** 2011-06-08

**Authors:** Steven W. Kembel, James F. Cahill

**Affiliations:** 1 Department of Biological Sciences, University of Alberta, Edmonton, Alberta, Canada; 2 Center for Ecology and Evolutionary Biology, University of Oregon, Eugene, Oregon, United States of America; University of Zurich, Switzerland

## Abstract

In this study, we used data from temperate grassland plant communities in Alberta, Canada to test two longstanding hypotheses in ecology: 1) that there has been correlated evolution of the leaves and roots of plants due to selection for an integrated whole-plant resource uptake strategy, and 2) that trait diversity in ecological communities is generated by adaptations to the conditions in different habitats. We tested the first hypothesis using phylogenetic comparative methods to test for evidence of correlated evolution of suites of leaf and root functional traits in these grasslands. There were consistent evolutionary correlations among traits related to plant resource uptake strategies within leaf tissues, and within root tissues. In contrast, there were inconsistent correlations between the traits of leaves and the traits of roots, suggesting different evolutionary pressures on the above and belowground components of plant morphology. To test the second hypothesis, we evaluated the relative importance of two components of trait diversity: within-community variation (species trait values relative to co-occurring species; α traits) and among-community variation (the average trait value in communities where species occur; β traits). Trait diversity was mostly explained by variation among co-occurring species, not among-communities. Additionally, there was a phylogenetic signal in the within-community trait values of species relative to co-occurring taxa, but not in their habitat associations or among-community trait variation. These results suggest that sorting of pre-existing trait variation into local communities can explain the leaf and root trait diversity in these grasslands.

## Introduction

Morphological and ecophysiological traits mediate interactions among plants, and knowledge of the functional traits of plants can provide important insights into the processes structuring plant communities [1]–[3]. Across a diverse set of plant taxa, there exist consistent tradeoffs governing the evolution of leaves [4]. However, only part of the plant's body lives above the soil surface, and our knowledge of root traits and their ecological and evolutionary relationships with leaf traits is limited [5] compared to our understanding of aboveground traits. In this study, we used data from temperate grassland plant communities to test two longstanding hypotheses in ecology: 1) that there has been correlated evolution of the leaves and roots of plants due to selection for an integrated whole-plant resource uptake strategy, and 2) that trait diversity in ecological communities is generated by adaptations to the conditions in different habitats.

A suite of correlated leaf traits known as the ‘leaf economics spectrum’ is found in plant species around the world; this spectrum separates ‘fast’ species that invest resources in short-lived leaves with a high expected rate of energetic return on investment from ‘slow’ species with longer-lived leaves with a slower expected rate of return [4]. ‘Fast’ species possess relatively large, fast growing leaves with short lifespan, high nitrogen concentration per unit mass, high specific leaf area (SLA; area per unit mass), and high instantaneous rates of respiration and photosynthesis [6], while ‘slow’ species possess the opposite set of traits. Similar patterns are found belowground, with ‘fast’-rooted species possessing thin, short-lived fine roots with high specific root length (SRL; length per unit mass), morphological plasticity, nitrogen concentrations and instantaneous rates of respiration and nutrient uptake, and low tissue density [7]–[10]. Despite the consistent trait correlations within leaves and within roots, relatively little is known about correlations between corresponding leaf and root traits within species [5].

Most adaptive explanations of leaf-root trait correlations have focused on the role of environmental conditions in different habitats giving rise to selection gradients for whole-plant resource uptake strategies [11]. Such strategies are typically believed to include changes to both root and shoot traits, leading to a prediction of correlations between corresponding leaf and root traits among species [11]. There has been mixed empirical evidence for leaf-root trait correlations [7], [12]–[15]. One potential explanation for these varied results is that existing studies have not accounted for phylogenetic relatedness when examining leaf-root trait correlations. Species are not statistically independent due to descent from a common ancestor [16], and phylogenetic comparative methods are required to test whether trait correlations represent correlated evolution due to selection or constraint versus an accident of history without an adaptive explanation [17]. While it has been hypothesized that leaf and root traits are correlated due to adaptive evolutionary responses to conditions in different habitats [1], [11], this hypothesis has never been directly tested in a phylogenetic comparative framework.

Related to the question of *whether* leaf and root traits are correlated is the question of *why* these traits are correlated. It has generally been assumed that among-community trait variation explains the majority of the observed trait correlations in plants [7], [12]–[15], although recent studies have contradicted this assumption [18]. Among-community trait correlations are generally thought to be caused by adaptation to abiotic conditions in different habitats [1], while within-community trait correlations are hypothesized to be due to biotic interactions or small-scale niche differentiation and character displacement [19]. The magnitude of trait variation among co-occurring species is often similar to or greater than trait variation among communities [20], but there have been few adaptive explanations of within-community trait variation [21].

The relative importance of within- and among-community trait variation in determining overall trait diversity among species will depend on whether trait diversity has arisen through ecological sorting of existing trait variation into communities, or through adaptive radiation after habitat colonization [22]. Following the terminology used in studies of species diversity [23], the within- and among-community components of trait variation have been referred to as the α and β components of trait variation, respectively [24]. The traits of an individual species can thus be thought of as the product of two components of trait variation: the ‘α trait’ (species trait values relative to co-occurring species) and the ‘β trait’ (the average trait value in communities where species occur) [24]. The ‘α first β throughout’ hypothesis [25] states that within-community niche differentiation (the ‘α niche’) tends to arise before among-habitat niche diversification (the ‘β niche’) as pre-existing trait variation is sorted by the environment [22], leading to a prediction of phylogenetic signal (a tendency for closely related species to resemble one another) in relative trait values within communities (‘α traits’) but not in trait variation among taxa living in different habitats (‘β traits’). Conversely, the ‘hierarchical diversification’ hypothesis states that colonization of different habitats precedes within-habitat adaptive diversification [26], leading to a prediction of phylogenetic signal in the traits of taxa living in different habitats (β traits) but not in the traits of co-occurring taxa (α traits). Variation in the way that different studies have defined ‘α traits’ and ‘β traits’ has made it difficult to test and evaluate the overall evidence for these competing hypotheses.

Our objectives in this study were to test the hypotheses that (1) leaf and root traits of plants have evolved in a correlated fashion as part of a whole-plant resource uptake strategy, and that (2) trait variation and inter-trait correlations reflect adaptations to local conditions and the environmental conditions in different habitats. We addressed these objectives by using phylogenetic comparative methods to test for evidence of correlated evolution of suites of leaf and root traits in temperate grassland plants, estimating the relative importance of variation in plant traits among and within communities of co-occurring species to explain overall patterns of trait diversity, and testing for phylogenetic signal in plant traits to understand the evolutionary origins of among- and within-community trait diversity.

## Methods

### Study system

Grassland communities at the northern fringe of the Great Plains in Alberta, Canada vary along major gradients of climate and soil, as well as along local environmental gradients within sites [27]. Mixedgrass communities dominate relatively xeric sites in south-eastern Alberta, with grasses such as needle-and-thread (*Hesperostipa comata* ssp. *comata*) and blue grama (*Bouteloua gracilis*) dominant [28], while the northern and western fringe of the grassland regions of the province are characterized by fescue-dominated communities with plains rough fescue (*Festuca hallii*) and porcupine grass (*Hesperostipa spartea*) among the dominant grasses [29], [30].

We measured plant species abundances and leaf and root traits at three sites located in two of the major grassland habitats in Alberta, fescue and mixedgrass grasslands. The Kinsella site (53°05′N, 111°33′W) is a rough fescue native grassland, and the Onefour (49°08′N, 110°31′W) and Hargrave sites (49°59′N, 110°02′W) are dry mixedgrass native grasslands. Lower precipitation and higher growing season temperatures, wind speeds, and evapotranspiration deficits at the Onefour and Hargrave sites result in an overall trend of expected greater drought stress and lower productivity at mixedgrass sites relative to fescue sites.

### Field sampling and trait measurement

During June and July of 2003 and 2004, we established eight to ten 20 m×20 m sampling plots at each site. Plots were distributed haphazardly along local topographic gradients in order to maximize the measured range of variation in plant community composition at each site. We recorded the identity of all vascular plant species present in ten 10×50 cm quadrats scattered randomly within each plot, which was sufficient for species accumulation curves within plots to saturate. Species abundances within each plot were defined as the percentage of quadrats in that plot in which a species was present. We defined communities as the species co-occurring in each 20 m×20 m plot.

We collected at least one healthy mature plant of each species in each plot for leaf and root trait measurement. Plants were collected in the morning, stored in plastic bags in a cooler and processed in the lab within 3 hours of collection. In order to allow measurement of fine root morphology, we excavated each plant with a portion of its root system intact by digging soil plugs measuring approximately 20 cm diameter and 20 cm deep, or deeper when necessary to obtain living fine root tissue from deep rooting or taprooted species.

Trait information was collected from each plant following published guidelines for leaf and root trait measurement [31]. For leaf traits, three mature leaves of each plant were scanned at 400 dpi for image analysis of one-sided projected leaf area using WinFOLIA software, and the thickness of the lamina of each leaf was measured to the nearest 0.1 mm using digital calipers. After measurement, leaves were dried for 72 hours at 70°C and weighed.

For root traits, after washing each plant over a sieve to remove soil, we carefully extracted a sample of the fine root system of each plant, ensuring that roots of surrounding plants were removed during washing. We defined fine roots as living roots with diameter <2 mm [31]. Fine roots were stored in a 70% ethanol solution and subsequently scanned at 800 dpi for image analysis of root length, volume, and average fine root diameter using WinRHIZO software. After scanning, fine roots were dried for 72 hours at 70°C and weighed.

In addition to direct measures of leaf size (one sided projected leaf area; cm^2^) and leaf thickness (mm) for each leaf, we estimated specific leaf area (SLA; leaf area per unit biomass; cm^2^/g) and leaf tissue density (leaf biomass per unit volume; mg/mm^3^), with leaf volume calculated as the product of leaf thickness and area. Similarly, we used direct measures of fine root sample length, volume, and diameters to estimate specific root length (SRL; root length per unit mass; m/g) and root tissue density (root mass per unit volume; mg /mm^3^) for each plant. Data on maximum average height for each species were obtained from a published flora [32].

### Phylogenetic data

**Figure 1:**
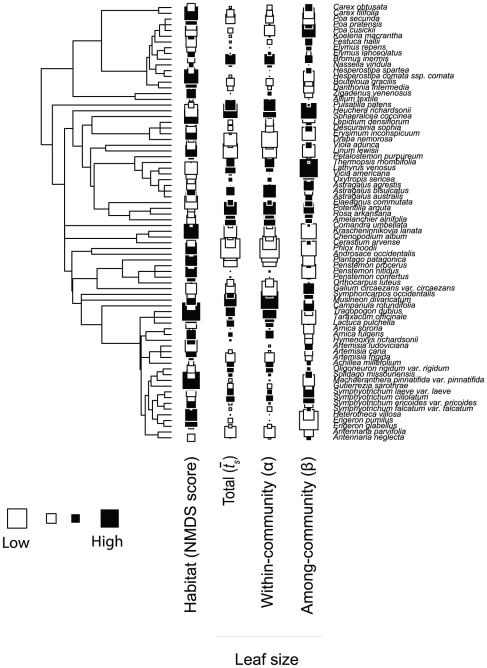
Hypothesized phylogenetic relationships and trait values for 76 plant species growing in Alberta grasslands. Symbols indicate relative values for species habitat associations (NMDS axis 1 score) and the total (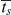), within-community (α), and among-community (β) components of leaf size variation (all values centered and scaled for visual comparison purposes). Branches are scaled proportional to estimated divergence times, with the root node (monocot – eudicot divergence) estimated at 139 million years ago.

We generated a phylogenetic hypothesis for the 76 species included in this study based on a published phylogenetic supertree of angiosperm families [33]. Species included in the present study were grafted onto the angiosperm strict consensus supertree at the crown clade age estimate for their family using Phylomatic [34]. Within-family phylogenetic relationships were resolved based on a variety of published phylogenetic trees for the families Asteraceae [35]–[37], Brassicaeae [38], Fabaceae [39], Poaceae [40], and Rosaceae [41]. Nodes in the resulting tree (Figure 1) that were not dated directly were spaced evenly between dated nodes to minimize tree-wide variance in branch lengths.

### Community composition and trait variation

Variation in the taxonomic species composition of all communities was summarized using a non-metric multidimensional scaling ordination (NMDS) based on the Bray-Curtis coefficient of community dissimilarity [42]. Ordinations were repeated 20 times from multiple random starting points, resulting in a two-dimensional solution (stress = 8.7, *P*<0.01 vs. Monte Carlo test with 999 runs).

To address hypotheses about the relative variation in traits within versus among communities, we used the method of trait-gradient analysis [24] to partition variation in leaf and roof traits into within-community (α trait) and among-community (β trait) components. Trait gradient analysis arranges communities along a gradient of mean community trait values, based on species' functional traits measured in each community. Following the notation of Ackerly and Cornwell [24], we defined *t_sp_* = the trait value of species *s* in plot *p*, *a_sp_* = the abundance of species *s* in plot *p*, *S* = the total number of species, and *P* = the total number of plots. Using these values, we estimated each species' mean trait value:
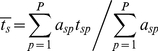
(1)and the mean trait value of plants in each plot weighted by the relative abundance of species within the plot:
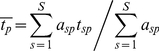
(2)



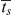
 is the total or species mean trait value that would be estimated for each species ignoring the relative magnitude of within- and among-community trait variation. Trait gradient analysis additively partitions this total trait value for each species into two components, the within-community component (α traits) and the among-community component (β traits). The α trait value for each species (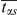) is a measure of that species' mean trait value relative to co-occurring species in plots where it occurs:
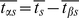
(3)The β trait value for each species (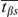) is a measure of mean location of each species along the trait gradient (the abundance-weighted mean of 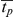 for plots containing the species):
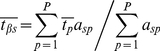
(4)Additively partitioning trait values in this way results in α and β trait values summing to the mean trait value for the species:
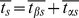
(5)


**Figure 2:**
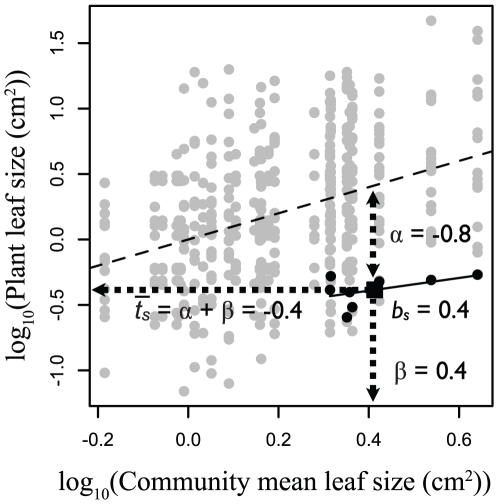
Trait gradient analysis of leaf size for 76 plant species growing in Alberta grasslands. Gray symbols are leaf size measured on 432 individual plants in 27 communities. Plants are arranged in order of increasing community mean leaf size (dashed line). Black symbols indicate leaf size of individual plants (circles) and the mean within-community (α) and among-community (β) leaf size (square) for *Galium boreale*. Dotted lines indicate mean leaf size of *Galium boreale* relative to co-occurring species (α = −0.8) and along the community mean leaf size gradient (β = 0.4), which sum to determine the mean or total leaf size observed for this species (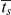 = 0.4). The solid line indicates the slope of within-species variation in leaf size in *Galium boreale* along the community trait gradient (*b_s_*).

We conducted trait gradient analyses for all measured leaf and roof traits based on the field-collected data on the abundances of species within sample plots, the leaf and root traits of the species collected in each plot, and height data collected from the literature (Figure 2). For <2% of plants we were unable to collect trait information for a species in the plot where it occurred due to local rarity or extremely deep taproots, in which case we substituted the mean trait values for that species based on collections from other plots at a site. All trait values were log_10_-transformed prior to analysis.

### Testing for correlated evolution of leaf and root traits

We tested for correlated evolution of leaf and root traits using phylogenetically independent contrasts to account for the non-independence of species due to their shared evolutionary history [16]. Contrasts in traits among descendants of each node in a phylogeny are statistically independent, and we considered statistically significant correlations between standardized contrasts as evidence for correlated evolution of traits [17]. Contrasts were calculated using Phylocom version 3.41 software [43]. Transforming all branch lengths to equal length improved contrast diagnostics (absolute contrasts and standard deviations were uncorrelated), supporting the use of equal branch lengths for all subsequent evolutionary analyses [17]. We tested for correlations between traits using species trait means. In addition to this ahistorical correlation, we repeated this correlation analysis using phylogenetically independent contrasts (PIC). PIC correlations were calculated based on standardized contrasts, calculating correlations through the origin and adjusting degrees of freedom [17].

### The relative importance of within- versus among-community trait variation

We calculated the variance in total trait values that could be explained by the within- and among-community components of trait variation using correlation analysis. To assess the magnitude of intraspecific trait variation we calculated the proportion of total variance in each trait occurring among individuals within species. We also estimated the pattern of intraspecific trait variation along the trait gradient using *b_s_*, the slope of *t_sp_* vs. 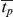 for each species, a measure of the relative shift in traits among plants within each species relative to the shift in mean trait values among communities. If trait values change at the same rate along the trait gradient within individual species as they do among communities, the expected value of *b_s_* is 1. We tested whether *b_s_* values for all species with at least three plants collected differed from 1 using a one-sample t-test.

### Phylogenetic signal in traits

To evaluate support for the ‘α first β throughout’ versus ‘hierarchical diversification’ hypotheses, we tested for phylogenetic signal in all traits using the *K* statistic [44], which measures the amount of variation in a quantitative trait relative to that expected under a Brownian motion model of trait evolution, with significance testing via comparison of the variance of standardized contrasts to random values obtained by shuffling trait data across the tips of the tree 999 times. Higher values of *K* indicate stronger phylogenetic signal, a tendency for close relatives to possess similar traits due to descent from a common ancestor [45]. The *K* statistic and associated *P*-value were calculated for all traits and the habitat associations of species (species scores on first axis of NMDS ordination) using Picante version 1.0 software [46].

## Results

### Community composition and community trait variation

**Figure 3:**
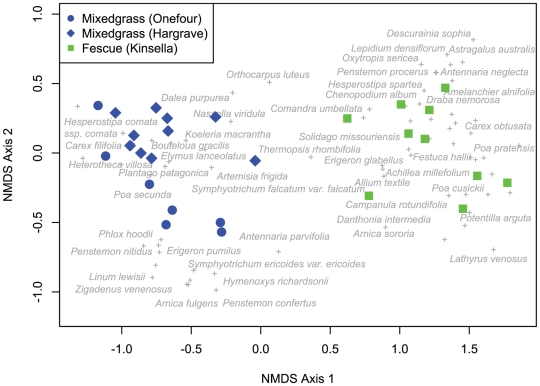
Results of a non-metric multidimensional scaling (NMDS) ordination for 27 communities in mixedgrass and fescue grasslands in Alberta. Solid symbols indicate the site and habitat type of individual communities (green = fescue site, blue = mixedgrass sites). Cross symbols indicate species scores; names of selected species are indicated in gray. The first axis of this ordination was used as a measure of the habitat affinity of species.

There were consistent differences in community composition between relatively xeric mixedgrass habitats and communities from more mesic fescue habitats (Figure 3). The first axis of the NMDS ordination separated plots from mixedgrass and fescue habitats, and species scores on this axis were thus used as a measure of the habitat affinity of individual species (Figure 3). While relatively few species were shared between these distinct habitat types (18 of 76 species were present in both habitat types), community scores on the first axis of the ordination were correlated with variation in plot-mean values of most traits (Table 1). The average plant in fescue communities possessed large, low density, high SLA leaves, and low SRL, low density, high diameter roots, compared to plants in mixedgrass communities (Table 1).

**Table 1:** Summary of trait-environment correlations and trait variation patterns for plant communities in mixedgrass and fescue grasslands in Alberta.

	Plot-mean trait value vs. plot NMDS axis 1 score correlation		*b*_s_ (intraspecific trait variation slope)			Trait variance within species (%)
Trait	r	*P*	Mean	SD	*P* (*t*-test, H_0_: *b_s_* = 1)	
Height	0.25	0.172				
SLA	0.61	0.001	0.86	1.37	0.553	12.6
Leaf size	0.84	<0.001	0.16	0.54	<0.001	1.6
Leaf thickness	−0.25	0.17	0.77	0.97	0.178	6.3
Leaf tissue density	−0.56	0.003	0.83	0.95	0.291	11.1
SRL	−0.51	0.008	1.2	1.7	0.5	11.8
Root tissue density	−0.39	0.047	0.83	0.97	0.314	14.6
Root diameter	0.4	0.042	0.57	1.37	0.078	7.5

Results include correlations between plot scores on non-metric multidimensional scaling (NMDS) ordination axis 1 and plot mean trait values for 27 communities in mixedgrass and fescue grasslands in Alberta, the slope of intraspecific trait variation (*b_s_*) for the 34 species occurring in at least 3 communities, and the proportion of total trait variance within species. Increasing plot NMDS axis 1 scores correspond to a transition from mixedgrass to fescue plant communities (*i.e.* a negative correlation indicates higher plot-mean trait values in mixedgrass communities).

### Testing for correlated evolution of leaf and root traits

**Figure 4:**
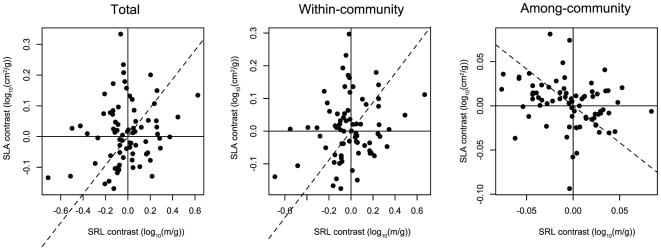
Correlations between phylogenetically independent contrasts of total (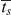), within-community (α), and among-community (β) components of trait variation for specific leaf area (SLA; cm^2^/g) versus specific root length (SRL; m/g). Dashed lines indicate estimated evolutionary correlation through the origin.

The among-community (β trait) component of interspecific trait variation was similar to patterns of variation in community mean trait values. The primary axis of interspecific leaf and root β trait variation separated species occurring in communities of relatively tall plants with low density tissues, high SLA, thin large leaves and thick high SRL roots, from species occurring in communities characterized by the opposite set of traits (Table 2, Figure 4).

**Table 2:** Summary of ahistorical and phylogenetically independent contrast (PIC) correlations among A) within-community (α), B) among-community (β) and C) total (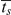) components of trait variation for 76 plant species (68 contrasts) in Alberta grasslands.

A. Within-community (α)	Height	SLA	Leaf size	Leaf thickness	Leaf tissue density	SRL	Root tissue density	Root diameter
Height		−0.09	**0.3**	0.14	−0.05	**−0.28**	0.03	**0.29**
SLA	−0.11		−0.21	**−0.35**	**−0.6**	**0.27**	0.2	**−0.42**
Leaf size	**0.46**	−0.13		**0.31**	−0.08	**−0.3**	−0.09	**0.37**
Leaf thickness	0.17	**−0.24**	0.3		**−0.53**	0.04	**−0.3**	0.11
Leaf tissue density	−0.04	**−0.58**	−0.14	−0.64		**−0.28**	0.08	**0.28**
SRL	**−0.32**	0.15	**−0.42**	−0.05	−0.07		**−0.4**	**−0.83**
Root tissue density	0.07	−0.03	−0.15	−0.31	**0.29**	**−0.28**		−0.15
Root diameter	**0.29**	−0.13	**0.52**	0.23	−0.09	**−0.8**	**−0.34**	

B. Among-community (β)	Height	SLA	Leaf size	Leaf thickness	Leaf tissue density	SRL	Root tissue density	Root diameter
Height		**0.4**	**0.69**	**−0.45**	0.11	−0.14	0.1	0.22
SLA	**0.26**		**0.78**	**−0.62**	**−0.45**	**−0.46**	**0.41**	−0.03
Leaf size	**0.71**	**0.7**		**−0.5**	**−0.41**	**−0.58**	0.17	**0.33**
Leaf thickness	**−0.41**	**−0.61**	**−0.52**		**−0.36**	0.08	**−0.27**	**0.26**
Leaf tissue density	0.2	**−0.48**	**−0.31**	**−0.34**		**0.54**	−0.03	**−0.34**
SRL	0.01	**−0.31**	**−0.4**	0.03	**0.43**		**−0.43**	**−0.46**
Root tissue density	−0.08	**0.3**	0	**−0.23**	0.03	**−0.32**		**−0.46**
Root diameter	0.21	−0.1	**0.26**	**0.25**	**−0.23**	**−0.42**	**−0.6**	

Cell contents are correlation coefficients. Below-diagonal values are ahistorical correlations, above-diagonal values are PIC correlations. Bold cells indicate correlations with *P*-value<0.05.

Since the majority of trait variation was among co-occurring species (92–98% of trait variation was within communities; Table 3), correlations among species mean trait values were driven by the within-community (α trait) component of trait variation. The main trends of leaf and root trait variation among co-occurring species in the field separated species with large leaves, thick roots and low SLA and SRL from those with the opposite set of traits, and separated species with thick leaves and low-density leaf and root tissue from those with the opposite set of traits (Table 2, Figure 4).

**Table 3:** Phylogenetic signal in within-community (α), among-community (β) and total (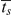) components of trait variation for 76 plant species in Alberta grasslands.

		Phylogenetic signal		Total variance explained
Component	Trait	*K*	*P*-value	R^2^
Within-community (α)	Height	0.30	0.003	0.98
	SLA	0.27	0.083	0.93
	Leaf size	0.38	0.001	0.92
	Leaf thickness	0.28	0.022	0.95
	Leaf tissue density	0.30	0.013	0.96
	SRL	0.23	0.113	0.97
	Root tissue density	0.28	0.003	0.94
	Root diameter	0.29	0.002	0.98
Among-community (β)	Height	0.26	0.065	0.20
	SLA	0.19	0.720	0.12
	Leaf size	0.20	0.519	0.18
	Leaf thickness	0.17	0.383	0.24
	Leaf tissue density	0.21	0.511	0.06
	SRL	0.18	0.996	0.06
	Root tissue density	0.18	0.500	0.17
	Root diameter	0.19	0.160	0.18
Total ( 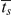 )	Height	0.31	0.003	
	SLA	0.24	0.306	
	Leaf size	0.39	0.001	
	Leaf thickness	0.26	0.032	
	Leaf tissue density	0.31	0.008	
	SRL	0.24	0.096	
	Root tissue density	0.30	0.001	
	Root diameter	0.29	0.002	

Total variance explained indicates the variation in species mean trait values (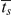) explained by each component.

Evolutionary correlations among leaf and root traits were generally similar to ahistorical correlations, although they tended to be higher in magnitude (Table 2). Ahistorical correlations between the within-community and total components of species leaf and root tissue densities disappeared after accounting for evolutionary relationships among species. There were no statistically significant evolutionary correlations between the within-community and among-community components of variation in individual traits.

### The relative importance of within- versus among-community trait variation

Although plot-mean trait values varied consistently among communities from mixedgrass versus fescue habitats (Table 1), the vast majority (92–98%) of community-level trait variation was within communities (among co-occurring species within each community; Table 3). Conversely, the vast majority of species-level trait variation was among species rather than within species; trait variation among individuals within species accounted for only 1.6% (leaf size) to 14.6% (root tissue density) of total trait variation (Table 1). Except for leaf size and to a lesser extent root diameter, the magnitude of trait variation among individual plants within species was equal in magnitude to the magnitude of plot-level trait variation among communities (one-sample t-tests of the mean value of *b_s_*; Table 1).

### Phylogenetic signal in traits

There was more phylogenetic signal than expected by chance for nearly all within-community components of trait variation, but none of the among-community components of trait variation (Table 3). Since total values of species traits were driven by within-community trait variation, these traits also generally exhibited non-random phylogenetic signal. Phylogenetic signal was strongest for architectural traits such as leaf size, leaf thickness, and root diameter. These traits were also the least variable within species and among communities (Table 1). There was no phylogenetic signal in species habitat associations (species NMDS scores; *K* = 0.15, *P* = 0.465).

## Discussion

### Testing for correlated evolution of leaf and root traits

We found limited support for the hypothesis that the leaf and root traits of plants have evolved in a correlated fashion as part of a whole-plant resource uptake strategy. Within leaves and within roots, trait correlations followed the patterns predicted by resource economics strategy theory [6], with high SLA leaves and high SRL roots tending to have lower tissue density and thickness, corresponding to an overall ‘fast’ resource uptake strategy. However, we detected complex relationships between corresponding leaf and root traits. SLA and SRL, leaf and root traits that have been proposed as indicators of the resource uptake strategies of species, were positively correlated within communities, but negatively correlated among communities (Table 2; Figure 4). Leaf and root tissue density were uncorrelated, while leaf and root thickness were positively correlated among communities but uncorrelated within communities. The inconsistency of these evolutionary correlations between leaf and root functional traits suggest that, rather than consistent selection for correlated leaf and root traits as part of a whole-plant, integrated resource uptake strategy in different habitats, there may be fundamentally different selective pressures and constraints on trait evolution above and belowground, and among versus within communities.

### The relative importance of within- versus among-community trait variation

Predictions of correlated above- and belowground trait evolution are often based on observations of trait variation along productivity or stress gradients, but differential above- and belowground effects of environmental constraints such as soil freezing and drought [15] or competition [47] may impose a different set of evolutionary pressures on leaf and root functional traits in different habitats. Environmental conditions in mesic fescue habitats include both a longer growing season with lower evapotranspiration deficits, leading to higher productivity above and belowground, but also cooler temperatures and greater tendency for soil freezing during the winter. This could lead to simultaneous selection for high SLA leaves to allow rapid resource uptake aboveground during the short growing season, and thick, low SRL roots to resist the mechanical effects of soil freezing.

Conversely, within-community trait variation likely incorporates much less environmental heterogeneity and biogeographic variation, given the much smaller spatial and environmental extent of individual communities [48], [49]. Within individual communities we did find evidence for stronger correlations between corresponding leaf and root traits such as SLA and SRL, suggesting that selection or constraint has led to concordant above- and belowground trait evolution at small scales. Environmental heterogeneity within local communities may also be driving the strong and consistent patterns of within-community trait correlations that we observed. Small patches of disturbed soil in these grasslands are often colonized by ruderal species with relatively high SLA and SRL [49], and this may have contributed to the within-community leaf-root correlations we observed [24]. It is interesting to note that a recent study that partitioned trait variation in forest vegetation into within- versus among-community components found a different pattern, with among-community trait correlations much stronger than within-community trait correlations [18]. Future studies that measure selection and partition trait variation into among and within community components across multiple spatial scales will be required to determine at which spatial scales environmental variation, versus fundamental constraints or selection, are responsible for correlated evolution of leaf and root traits, and the relative importance of within- versus among-community trait correlations to explain plant trait strategies.

Traits changed as much within species as they did among community mean trait values along the trait gradient for most traits (t-test of *b_s_* values; Table 1), indicating that differences in community-mean trait values were driven by both intraspecific variation and species turnover, despite the small proportion of species shared between the two major habitat types we studied, and the relatively small proportion of trait variation within species. This result is similar to a recent study of leaf traits in tropical forests, which also found substantial trait variation within communities and species [21]. While it has been argued that common garden experiments are needed to understand the adaptive significance of trait correlations due to the potentially confounding effects of intraspecific variation [50], our findings indicate that trait variation within species and among communities, rather than being an artefact that needs to be eliminated by growth under common conditions, can be quantified and may help explain patterns of trait differentiation among habitats and species.

### Phylogenetic signal in traits

Many closely related species pairs possessed similar trait values relative to co-occurring species (α traits), but with one species occurring primarily in mixedgrass habitats and the other in fescue-dominated grasslands (high phylogenetic signal in within-community traits but no signal in habitat associations; Table 1, Figure 1). Part of the difficulty reconciling previous studies of α and β niches and traits has been the ways these traits have been defined. Traits are often used as surrogate measures of the niches occupied by species, but different studies have often used the same traits as measures of both α and β niches. For example, SLA has been used both as an indicator of the α niche [25] and β niche [22]. While the relative magnitude of α and β trait variation will clearly depend on the spatial scale and environmental extent used to define communities [24], partitioning individual traits into these components provides a way to quantify within and among community variation without assumptions about which traits are the best indicators of functional strategies at that scale.

By measuring the different components of trait diversity directly, we found evidence that α traits and niches are less evolutionarily labile than β traits, and that all traits vary at both α and β scales. Our results support the ‘α first β throughout’ model of trait diversification [25], but do not support the hypothesis that α niches are more evolutionarily labile than β niches [26], [51]. Different clades appear to occupy characteristic niches within the grassland communities we studied, and there is phylogenetic conservatism of traits relative to co-occurring species, but sorting of species into different habitats seems to have occurred repeatedly throughout evolutionary time, as evidenced by the lack of phylogenetic signal in habitat associations and trait syndromes in different habitats.

### The ecological and evolutionary origins of grassland plant trait diversity

Ecological sorting of trait diversity that arose deep in evolutionary time appears to explain much of the present-day trait diversity in the temperate grasslands we studied. At the same time, our results are consistent with ongoing selection for trait differentiation within communities via character displacement, plasticity or adaptive change in traits [19]. Communities dominated by species present in these grasslands have been widespread in the Great Plains region of North America since the late Miocene [52], but most of the trait variation we observed appears to be among clades that diverged earlier than the late Miocene, for example among families such as the Poaceae, Fabaceae and Asteraceae, each of which had a characteristic set of traits relative to co-occurring species (Figure 1). This is similar to patterns observed at much broader spatial scales, for example in the grasses, whose radiation and functional diversification appears to have predated the origin of the northern temperate grassland biome [53], [54], and our results along with other recent studies [22], [25] suggest that this ‘α first β throughout’ pattern of trait diversification may be a widespread phenomenon in plant communities. However, as with all ecological studies our findings are scale-dependent, and the generality of our findings will need to be tested by future studies at a wider range of spatial and environmental scales. We defined communities as organisms co-occurring at a relatively small spatial scale, and studied grassland communities in a single region. We hypothesize that the magnitude of among-community trait variation relative to within-community variation will increase when studied at larger scales and across broader environmental gradients, as greater variation in species traits and community structure are encountered.

In summary, we found limited support for the hypothesis that leaf and root traits of plants have evolved in a correlated fashion as part of a whole-plant resource uptake strategy. Trait correlations within leaves and within roots followed the patterns predicted by leaf resource economics theory, but there were complex and inconsistent relationships between corresponding leaf and root traits. We also found that the diversity of leaf and root traits among species in Alberta grasslands cannot be explained solely by selection for whole-plant resource uptake strategies in different environments, or by contemporary ecological interactions. Our results indicate the importance of considering evolutionary history and biogeography when studying trait diversity in ecological communities, but also raise the question of what has generated and maintained consistent patterns of within-community trait diversity throughout time. Several explanations are possible including fundamental constraints on trait evolution [55], or ongoing and consistent ecological selection pressures on functional traits and plant strategies [1]. Disentangling the impact of these different processes on contemporary trait diversity will require a much broader synthetic approach that incorporates phylogenetic information, explicit models of trait evolution and community assembly, and a more biogeographic and evolutionary perspective to explain ecological species, trait and phylogenetic diversity.
